# Determinants of under-five mortality in Sri Lanka: A multilevel analysis of 2016 Sri Lankan DHS data

**DOI:** 10.1371/journal.pone.0291246

**Published:** 2023-09-08

**Authors:** Kaludura Anupama Seuwandi Thabrew, Marina Roshini Sooriyarachchi, Dushantha Nalin K. Jayakody

**Affiliations:** 1 Faculty of Arts & Science, Sri Lanka Technological Campus, Padukka, Sri Lanka; 2 Department of Statistics, Faculty of Science, University of Colombo, Colombo, Sri Lanka; 3 School of Engineering, Sri Lanka Technological Campus, Padukka, Sri Lanka; 4 TECHLAB—Centro de Investigação em Tecnologias, Universidade Autónoma de Lisboa, Lisbon, Portugal; University of the Punjab, PAKISTAN

## Abstract

Under-five mortality (U5M) is considered a major public health issue directly impacts a country’s development. This study analyzed the prognostic factors of U5M in Sri Lanka using data from the 2016 Demographic and Health Survey (DHS) of 8123 children. The study employed both a binary logistic regression model (BLRM) and a binary logistic random intercept multilevel model (BLRIMM) and compared the accuracy of each model’s prediction percentage. The results showed that the BLRIMM had a higher correct prediction percentage (98.67%) compared to the BLRM (98.31%). The study found that children who were not breastfed (Odds Ratio (OR) = 116.74, 95% Confidence Interval (CI) = 62.97–216.41), were part of multiple births (OR = 3.73, 95% CI = 1.21–11.51), did not have a normal delivery (OR = 1.86, 95% CI = 1.11–3.12), were born to mothers who had experienced previous miscarriages or child loss (OR = 2.27, 95% CI = 1.26–4.11), and were born to mothers with a higher Body Mass Index (BMI) (OR = 1.05, 95% CI = 1.003–1.10) had higher odds of U5M. The odds of U5M were found to be lower among Buddhists (OR = 0.06, 95% CI = 0.01–0.50), Hindus (OR = 0.05, 95% CI = 0.01–0.46), and Roman Catholics (OR = 0.032, 95% CI = 0.003–0.307) compared to the "Other Religions" category in the dataset. The estimated covariance parameter of the random intercept (0.8231, p-value = 0.0405) indicated significant unobserved cluster-level variation in U5M. The study’s results emphasize the importance of addressing religion related differences of U5M and improving maternal education regarding healthy lifestyle, proper food intake, the significance of breastfeeding, safe delivery methods, safety measures during pregnancy and childbirth in cases of multiple births, and proper child care after birth.

## Introduction

Under-five Mortality (U5M), which is defined as the death of a child between birth and the fifth birthday, is considered a critical public health issue. In 2021, global U5M was recorded at 5 million deaths, and tragically, most of these deaths were caused by preventable or treatable conditions [1]. After recognizing the importance of reducing U5M rates, in 2000, the United Nations set a Millennium Development Goal to reduce U5M rates to 30 per 1,000 live births, but this target was not achieved globally [2]. In 2016, the Sustainable Development Goals were established, with a goal of reducing under-5 mortality to at least 25 deaths per 1,000 live births and reducing neonatal mortality to at least 12 deaths per 1,000 live births, to be achieved by 2030. The goal also included ending preventable deaths of neonates and children under five years old [3]. With advances in technology and improvements in the medical field, the global U5M rate has declined by 59% from 1990 to 2021 [4]. Substantial progress has been made through high impact interventions such as postnatal care, access to nutrition and micronutrients, skilled attendants during birth, immunizations, and expanded access to safe water, sanitation, and hygiene [5]. Despite these efforts, there are still some countries struggling to reduce U5M rates.

Sri Lanka is ranked 178^th^ out of 241 countries in the 2020 list of countries with the highest to lowest U5M rate, with a rate of 6.9 deaths per 1,000 live births [6]. Literature [7] provides a map showing the percentage distribution of U5M rates in South Asia in 2020, and according to the map, Sri Lanka has a lower U5M rate compared to many other South Asian countries. However, as stated by Roser and Ritchie [8], it is evident that there are still significant disparities in U5M rates among developed and developing nations globally. Hence, the importance of studying the patterns and variations in U5M rates is high, as it directly affects the health of children and the well-being of populations. A decline in U5M rates is crucial for increasing life expectancy [8] and improving the Human Development Index (HDI), which is a widely accepted indicator of social development and a good marker of a country’s development, especially in low and middle-income countries [9].

### Literature review

#### Foreign literature

Considering factors associated with U5M in different countries around the world, many research studies have been conducted and various significant factors of U5M have been identified in different regions.

In a study conducted in South Asia and Sub-Saharan Africa [10], the authors used a multilevel approach to analyze U5M in these regions. The study analyzed the effects of socioeconomic characteristics at the individual, household, and cluster levels on both child mortality and malnutrition. The conclusion was that the determinants of malnutrition and child mortality differ significantly, and access to health infrastructure is significantly associated with child mortality. In 2003, Mosley and Chen [11] introduced an analytical framework for studying the socio-economic determinants of child mortality in developing countries. According to the framework, these determinants can be grouped into five categories: personal illness control, injury, nutrient deficiency, environmental contamination, and maternal factors. A study in Jordan [12] found that infant and U5M rates can be reduced by providing safe drinking water. In Nepal [13], a study was conducted to investigate the association between antenatal iron-folic acid supplements and U5M and found that the supplements significantly reduced the odds of U5M. In Punjab, India [14], a predictive model was developed to examine the significant predictors of U5M. The results showed that factors such as the number of children, access to clean drinking water, stunting, improved sanitation, mother’s education, skilled birth attendant, premature birth, household wealth status, and mother’s age at childbirth were significant predictors of U5M. Studies conducted in Bangladesh [15] and India [16] emphasized the importance of getting a higher dose of the Tetanus Toxoid vaccine. These studies revealed that protection against neonatal tetanus with just one dose of the vaccine is not satisfactory and increasing the dose to at least two doses would result in a gain in neonatal survival. A study in Nepal [17] found that children of mothers who did not use contraceptives, did not receive the Tetanus Toxoid (TT) vaccine during pregnancy, and had a previous child death were at higher odds of dying before five years old. A study in Ethiopia [18] found that the income of mothers, source of drinking water, breastfeeding status, type of birth, family size, and birth interval were significant determinants of U5M. Another study in Bangladesh [19] concluded that factors such as contraceptive use by mothers, having other children under 5 years old, previous death of a sibling, birth interval, and birth rank were associated with U5M. In Ethiopia [20], a study was conducted to investigate factors affecting U5M using a multilevel negative binomial model for data analysis. The results showed that residents of Addis Ababa, having a preceding birth interval, delivering at a health institution, and the mother’s education reduced the odds of U5M. Conversely, factors such as childhood diarrhea, multiple births, employment, age of mother at first birth, and being a female household head were associated with higher odds of U5M.

#### Local literature

There have been only a few studies conducted in Sri Lanka to examine the determinants of U5M. One study, conducted in 1980, found a strong relationship between child mortality levels and the availability of toilet facilities [21]. In another study, conducted in 1983 in Sri Lanka, factors such as type of toilet facility, mother’s age at birth, birth order, sex, ethnicity, urban/rural/estate residence, time period of birth, and the education levels of the mother and father were found to be strongly associated with U5M [22]. A study conducted in Sri Lanka in 2011 examined the factors contributing to the reduction of infant mortality in the country and concluded that the infant mortality rate had been reduced due to high immunization coverage of pregnant mothers and infants, improved female literacy rates, the elimination of neonatal tetanus, free healthcare, and increased institutional deliveries [23]. Most of these studies were conducted before 1990, and their results may not be valid today due to significant improvements and changes in many fields, including medicine. Additionally, the trends and features of the population have changed over time, due to changes in factors such as population size, density, age distribution, natality, mortality, growth, and fertility [24]. A 2021 study on child mortality in Sri Lanka only considered household characteristics and did not take into account the delivery method, type of birth, gender of the child, or the mother’s characteristics, such as seeking antenatal care (ANC) and taking antenatal supplements [25]. The study results showed that place of residence, education level of the household head, sources of drinking water, and ethnicity were significantly associated with U5M. None of these studies have examined the variability of U5M across geographical clusters in the country or incorporated the cluster effect into their analysis. The elements within one cluster tend to be more similar to each other than to elements in other clusters. Multilevel modeling is important in the presence of clusters to prevent biased parameters and standard errors and to improve predictions, as in this research.

The primary objective of this study is to identify the prognostic factors associated with U5M in Sri Lanka and to determine the effect of these factors in predicting U5M. The goal of this study is to use the results to assist relevant authorities in making informed decisions, provide a foundation for policy formulation, aid in planning effective health programs, and educate society about the prognostic factors, so that efforts can be made to reduce them starting at the household level.

## Materials, theory and methods

### Data

The study utilized data from the most recent Demographic and Health Survey (DHS) conducted in Sri Lanka in 2016 by the Department of Census and Statistics (DCS). No previous studies on U5M have been conducted using the data set considered in this research. The survey employed a two-stage stratified sampling design. In the first stage of the design, 2,500 census blocks/clusters were selected as the primary sampling units (PSUs). From these selected PSUs, 10–12 housing units were selected as the secondary sampling units (SSUs). This study used data from 8,123 under-five children from the selected SSUs who were either dead or alive at the time of the survey.

### Ethical approval

The necessary authorization and ethical approval for this study were obtained from the Sri Lanka DCS. Since the research utilized secondary data, obtaining consent from the participants was not necessary. All procedures followed the appropriate guidelines and regulations.

### Variables

The dependent variable used in this study was the living status of a child born within the five years prior to the time of the survey, which is a binary variable. The independent variables used in this study were categorized as child variables, mother variables, household variables, and community variables, and they were selected based on the reviewed literature. Detailed descriptions of all variables used in this study are presented in Table 1.

**Table 1:** Details of variables.

Variable	Code
**Child Variables**	
Living Status (Dependent Variable)	0- Living
	1- Dead
Birth Order	1- 1^st^
	2- 2^nd^ or Above
Child was breastfed	1- Yes
	2- No
Gender of the Child	1- Male
	2- Female
Place of Delivery	1- Government Hospitals & Specialized Service^1^
	2- Other Government Hospitals^2^
	3- Private Hospital/ Estate Maternity Home
	4- Home
	5- While Going to the Hospital/ Other
Type of Birth	1- Single
	2- Multiple
Type of Delivery	1- Normal Delivery
	2- Other Delivery Types^3^
**Mother Variables**	
Ethnicity	1- Sinhala
	2- Sri Lanka Tamil
	3- Indian Tamil
	4- Sri Lanka Moor/ Muslim
	5- Malay/ Burger
Religion	1- Buddhist
	2- Hindu
	3- Islam
	4- Roman Catholic
	5- Other Christian
	6- Other Religions ^4^
Marital Status	1- Married
	2- Other
Mother’s Education	1- Primary-School or less
	2- Secondary-School
	3- Higher
Husband’s/ Partner’s Education	1- Primary-School or less
	2- Secondary-School
	3- Higher
Mothers Age at First Marriage (Continuous Variable)	-
Mother’s Age (Continuous Variable)	-
Mother’s Body Mass Index (BMI) (Continuous Variable)	-
Use of Contraceptive Methods	1- Yes
	2- No
Previous Death/ Miscarriage of a Child	1- Yes
	2- No
Status of Antenatal Visits	1- Yes
	2- No
Pregnancy TT Received	1- Yes
	2- No
Times Pregnancy TT Received	0- None
	1- Once
	2- Twice or More
Took Calcium Pills	1- Yes
	2- No
Took Folic Acid Pills	1- Yes
	2- No
Took Iron Pills	1- Yes
	2- No
Took Other Vitamins	1- Yes
	2- No
Took Worm Treatments	1- Yes
	2- No
Mother’s Working Status	1- Yes
	2- No
Mother’s Experience with Domestic Violence	1- High
	2- Medium
	3- Low
**Household Variables**	
Household Size	2- Two
	3- Three
	4- Four
	5- Five or More
Source of Drinking Water	1- Pipe Born Water
	2- Well
	3- Other
Type of Sanitation Facility	1- Flush/Pour Flush Toilet
	2- Pit Latrine
	3- Other
Wealth Index Quintile	1- Lowest
	2- Second
	3- Middle
	4- Fourth
	5- Highest
**Community Variables**	
Type of Residence	1- Urban
	2- Rural
	3- Estate
Province	1- Western Province
	2- Central Province
	3- Southern Province
	4- Northern Province
	5- Eastern Province
	6- North Western Province
	7- North Central Province
	8- Uva Province
	9- Sabaragamuwa Province
**Identifier Variables**	
Cluster number	
Kids Number	

^1^ Government Hospitals & Specialized Service includes teaching hospitals, provincial general hospitals and base hospitals

^2^ Other Government Hospitals includes district hospitals, peripheral hospitals, rural hospitals and maternity homes

^3^ Other delivery methods include delivery using caesarian, forceps and vacuum method.

^4^ Other Religions represents religions followed by Sri Lankans, such as Baháʼí Faith, Sikhism and Atheist other than the main religions mentioned in the categories.

### Theory

#### Multilevel logistic regression model

Initially, the theory behind the multilevel binary logistic regression model for multilevel binary response data is considered. It is often not possible to deal with continuous response variables, and binary and categorical responses are a common phenomenon in many areas of study. If the response has only two levels, it is considered a binary or dichotomous response variable.

#### Single-level model (A binary logistic regression model)

According to Browne [26] consider a single level logistic regression model with single explanatory variable *x*_*i*_. The binary (0, 1) response for the *i*^th^ unit is denoted by *y*_*i*_. Let the probability that *y*_*i*_ = 1 be *π*_*i*_.

A general model for binary response data is:

$$f\left(\pi_{i}\right)=\;\beta_{0}+\beta_{1}x_{i},$$ (1)

where *f*(*π*_*i*_) is some transformation of *π*_*i*_, called the link function. Popular choices for the link function are logit link, probit link or complementary log-log link (clog-log link). The logit transformation tends to be most widely used, mainly because the exponentiated coefficients from logit model can be interpreted as odds ratios [26]. Therefore, in this research the logit link function which is shown as below is considered.

$$f\left(\pi_{i}\right)=\;\text{log}\left(\frac{\pi_{i}}{1-\pi_{i}}\right),$$ (2)

where the quantity $\left(\frac{\pi_{i}}{1-\pi_{i}}\right)$ is the odds that *y*_*i*_ = 1.

Therefore, logit model takes the form:

$$logit\left(\pi_{i}\right)=log\left(\frac{\pi_{i}}{1-\pi_{i}}\right)=\;\beta_{0}+\beta_{1}x_{i}.$$ (3)

#### Two-level model (Binary logistic random intercept multilevel model)

Single level logistic regression model is now extended to two level random intercept or variance components model that allows the overall probability of response of interest to vary across the second level (clusters) [27].

Now the binary response is:

$$y_{ij}=\left\{\begin{array}{ll} 1, & if\;i^{th}\;observation\;in\;the\;j^{th}\;cluster\;has\;the\;response\;of\;interest\\ 0, & otherwise. \end{array}\right.$$

Similarly, a *j* subscript is added to the proportion so that *π*_*ij*_ = Pr(*y*_*ij*_ = 1).

Suppose there is a single explanatory variable, *x*_*ij*_, measured at the 1^st^ level, then the single-level model can be extended to a two-level random intercept model such as follows:

$$logit\left(\pi_{ij}\right)=\beta_{0j}+\beta_{1}x_{ij,}$$

$$\beta_{0j}=\beta_{0}+u_{0j}.$$ (4)

For a random intercept model for a binary response, the intercept consists of two terms: a fixed component *β*_0_ and a cluster specific component, the random effect *u*_0*j*_. Here it is assumed that the *u*_0*j*_ follows a Normal distribution with mean zero and variance $\sigma_{u0}^{2}$ (i.e. $u_{0j}\sim \;N\left(0,\;\;\sigma_{u0}^{2}\right)$).

#### Comparison of proportions based on the normal approximation

Let P_1_ = The success probability in the first population and let P_2_ = The success probability in the second population

To compare the two probabilities, the hypotheses of Null (H_0_) and Alternative (H_1_) for a one sided hypothesis can be given by,

Need to test H_0_: P_2_ = P_1_ versus

H_1_: P_2_ > P_1_

The probabilities P_1_ and P_2_ can be estimated by the sample proportions p_1_ and p_2._ The difference in the observed proportions p_2−_p_1_ has

$$mean=\;P_{2}-\;P_{1}$$ (5)

and

$$Variance=\;\frac{P_{2}\;\left(1-\;P_{2}\right)}{F_{2.}}+\;\frac{P_{1}\;\left(1-\;P_{1}\right)}{F_{1.}},$$ (6)

where F_1._and F_2._ are the row totals corresponding to the two samples of interest.

Since we do not know the values of P_1_ and P_2_, we use p_1_ and p_2_ to estimate the variance.

Under H_0_: P_2_ = P_1_ (= P say)

The overall probability of success can be estimated by the overall sample proportion of success

$$p=\frac{\left(F_{1.}p_{1}+F_{2.}p_{2}\right)}{F_{1.}+F_{2.}}.$$ (7)

Thus under H_0_ the variance of p_2_ – p_1_ can be estimated by p(1-p) [1/F_1._ + 1/F_2._]

Thus under H_0_, a reasonable test for comparing the two proportions can be based on the approximate normality of the test statistic,

$$Z=\frac{p_{2}-\;p_{1}}{\sqrt{\text{p}\left(1-\text{p}\right)\left(\frac{1}{F_{1.}}+\frac{1}{F_{2.}}\right)}}.$$ (8)

[Note: The approximation is valid for large samples (i.e. large F_1._ and F_2._) and improves as p_2_, p_1⇢_ 0.5].

### Statistical analysis

Initially, the univariate and bivariate analysis pertaining to the descriptive part of the study variables were explored. Then, the presence of multicollinearity between the independent variables was checked using the variance inflation factor (VIF) [28]. A Binary Logistic Regression Model (BLRM) was fitted using a forward selection method [29], followed by a Binary Logistic Random Intercept Multilevel Model (BLRIMM) [27] where the significant variables from the BLRM were used in the latter model. This method was adopted as the dataset had both categorical and continuous variables and the variable selection for the BLRIMM could be done effectively for both variable types using the BLRM.

The importance of involving a multilevel model in the model building is due to the clustered nature of the data, as explained previously. Therefore, it is vital to examine the significance of these cluster terms and how important it is to include these in the model.

For validation purposes, the predicted probabilities of U5M in both the BLRM and the BLRIMM had to be tested against one or more cut-points. The usual cut-point of 0.5 was not appropriate here, as there were far fewer deaths than those alive. For the former model, the cut-point was taken as 0.0767, and for the latter model, it was taken as 0.15, based on the ROC curve and the AUC for different cut-points [30]. Whenever these predicted probabilities were equal to or greater than the cut-point, the fitted value was taken as 1, counting it as a death. Predicted probabilities that were less than the cut-point were coded as 0, counting those observations as alive. Then, 2x2 classification tables of the predicted outcomes and observed outcomes were created for both the BLRM and the BLRIMM, and the percentage of correctly predicted observations were compared to examine which model predicted more observations correctly. A test to compare the proportions based on the normal approximation was used to test the significance of the difference between the correctly predicted percentages by the two models.

Sample weights were used in the analysis. P-values less than 0.05 were considered statistically significant, and 95% confidence intervals were considered in this study.

## Results

**Fig 1:**
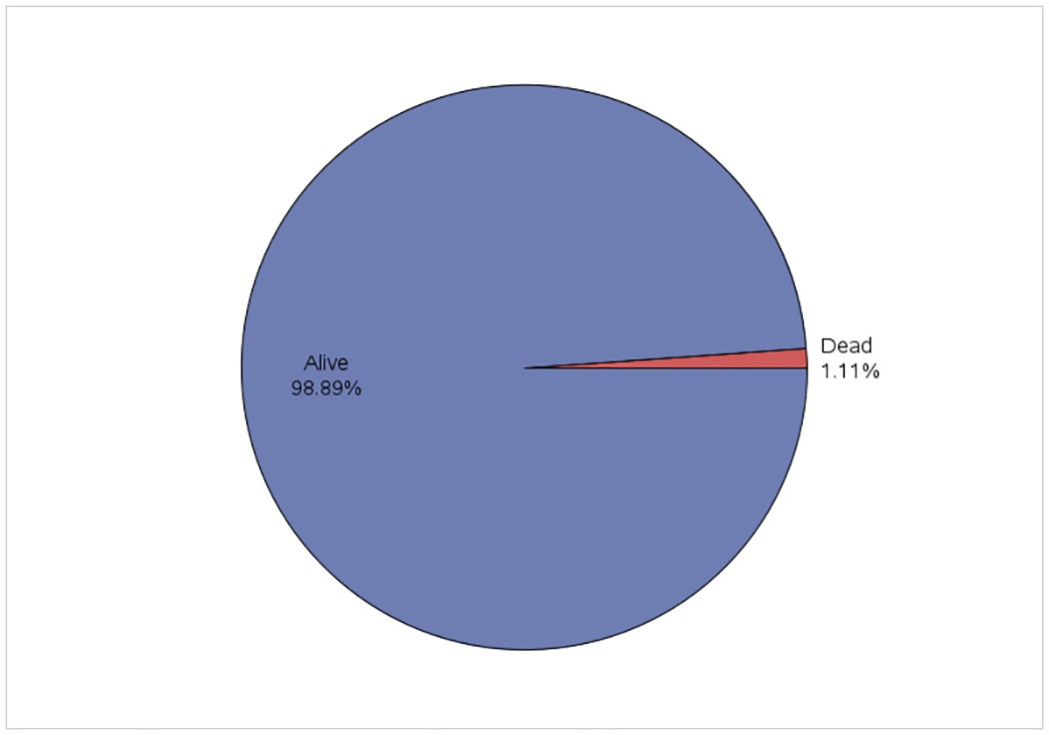
Distribution of living status of under five children.

The distribution of the "Living Status" in the study sample is depicted in Fig 1 and the results show that the rate of U5M is 1.11% (11 deaths per thousand live births).

### Descriptive statistics

Table 2 presents the frequency and proportion distribution of the independent categorical variables used in this study across the living status of under-five children. The results show that the prevalence of U5M is high among children who have a birth order of 2 or higher (1.20%), who were not breastfed (37.07%), who were born as part of a multiple birth (4.05%), and who were born using other delivery methods (1.68%). Children who were born at home (8.33%) and male children (1.17%) also showed high prevalence of U5M. Additionally, the U5M rates were high among children whose mothers were Sri Lankan Tamil (1.66%), belonged to other Christian categories (8.33%), and were married (1.11%). Furthermore, the prevalence of U5M was high among children whose mothers had only up to secondary school education (1.19%) and whose mothers’ husbands or partners had higher education (1.45%). The prevalence of U5M was also high among children whose mothers did not use any contraceptive method (2.47%), had a previous death or miscarriage of a child (2.14%), did not go for antenatal visits (1.52%), received only one pregnancy TT vaccine (1.58%), and did not take calcium pills (1.20%) or other vitamins, but took folic acid pills (1.12%), iron pills (1.11%), or received worm treatments (1.11%). Additionally, higher U5M was shown among children whose mothers were working (1.25%) and experienced low rates of domestic violence (1.12%). High under-five deaths were reported among children who were born into households with a size of 3 (2.22%), had pipe-borne water as the source of drinking water (1.21%), had pit latrines (1.20%), and belonged to the lowest wealth index quintile (1.46%). Furthermore, the prevalence of U5M among children from the estate sector (1.71%) and the Eastern province (1.95%) was high.

**Table 2:** Distribution of independent categorical variables with the living status.

Variable	Overall	Living Status of the Child	
	n = 8123	Alive	Dead
	N(%)	N(%)	N(%)
**Child Variables**			
Birth Order			
1st	3128 (38.51%)	3098 (99.04%)	30 (0.96%)
2nd or Above	4995 (61.49%)	4935 (98.80%)	60 (1.20%)
Child was Breastfed			
Yes	8007 (98.57%)	7960 (99.41%)	47 (0.59%)
No	116 (1.43%)	73 (62.93%)	43 (37.07%)
Type of Birth			
Single	7975 (98.18%)	7891 (98.95%)	84 (1.05%)
Multiple	148 (1.82%)	142 (95.95%)	6 (4.05%)
Type of Delivery			
Normal Delivery	5626 (69.26%)	5578 (99.15%)	48 (0.85%)
Other Delivery Types	2497 (30.74%)	2455 (98.32%)	42 (1.68%)
Place of Delivery			
Government Hospital & Specialized Service	6703 (82.52%)	6629 (98.90%)	74 (1.10%)
Other Government Hospital	995 (12.25%)	984 (98.89%)	11 (1.11%)
Private Hospital/ Estate Maternity Home	390 (4.8%)	387 (99.23%)	3 (0.77%)
Home	12 (0.15%)	11 (91.67%)	1 (8.33%)
While going to the Hospital/ Other	23 (0.28%)	22 (95.65%)	1 (4.35%)
Gender of the Child			
Male	4181 (51.47%)	4132 (98.83%)	49 (1.17%)
Female	3942 (48.53%)	3901 (98.96%)	41 (1.04%)
**Mother Variables**			
Ethnicity			
Sinhala	4889 (60.19%)	4845 (99.10%)	44 (0.90%)
Sri Lankan Tamil	1502 (18.49%)	1477 (98.34%)	25 (1.66%)
Indian Tamil	1001 (12.32%)	986 (98.50%)	15 (1.50%)
Sri Lankan Moor/Muslim	617 (7.6%)	612 (99.19%)	5 (0.81%)
Malay/Burger	114 (1.4%)	113 (99.12%)	1 (0.88%)
Religion			
Buddhist	5228 (64.36%)	5180 (99.08%)	48 (0.92%)
Hindu	1694 (20.85%)	1671 (98.64%)	23 (1.36%)
Islam	256 (3.15%)	251 (98.05%)	5 (1.95%)
Roman Catholic	913 (11.24%)	901 (98.69%)	12 (1.31%)
Other Christian	12 (0.15%)	11 (91.67%)	1 (8.33%)
Other Religions	20 (0.25%)	19 (95.00%)	1 (5.00%)
Marital Status			
Married	7984 (98.29%)	7895 (98.89%)	89 (1.11%)
Other	139 (1.71%)	138 (99.28%)	1 (0.72%)
Mother’s Education			
Primary School or Less	414 (5.1%)	410 (99.03%)	4 (0.97%)
Secondary School	5398 (66.45%)	5334 (98.81%)	64 (1.19%)
Higher	2311 (28.45%)	2289 (99.05%)	22 (0.95%)
Husband’s/ Partner’s Education			
Primary School or Less	554 (6.82%)	548 (98.92%)	6 (1.08%)
Secondary School	6120 (75.34%)	6057 (98.99%)	63 (1.03%)
Higher	1449 (17.84%)	1428 (98.55%)	21 (1.45%)
Use of Contraceptive Methods			
No	809 (9.96%)	789 (97.53%)	20 (2.47%)
Yes	7314 (90.04%)	7244 (99.04%)	70 (0.96%)
Previous Death/ Miscarriage of a Child			
Yes	1120 (13.79%)	1096 (97.86%)	24 (2.14%)
No	7003 (86.21%)	6937 (99.06%)	66 (0.94%)
Status of Antenatal Visits			
Yes	8057 (99.19%)	7968 (98.90%)	89 (1.10%)
No	66 (0.81%)	65 (98.48%)	1 (1.52%)
Pregnancy TT Received			
Yes	7880 (97.01%)	7791 (98.87%)	89 (1.13%)
No	243 (2.99%)	242 (99.59%)	1 (0.41%)
Times Pregnancy TT Received			
None	425 (5.23%)	424 (99.59%)	1 (0.41%)
Once	4606 (56.7%)	4533 (98.42%)	73 (1.58%)
Twice or More	3274 (40.31%)	3258 (99.51%)	16 (0.49%)
Took Calcium Pills			
Yes	8040 (98.98%)	7951 (98.89%)	89 (1.11%)
No	83 (1.02%)	82 (98.80%)	1 (1.20%)
Took Folic Acid Pills			
Yes	8061 (99.24%)	7971 (98.88%)	90 (1.12%)
No	62 (0.76%)	62 (100.00%)	0 (0.00%)
Took Iron Pills			
Yes	8009 (98.6%)	7920 (98.89%)	89 (1.11%)
No	114 (1.4%)	113 (99.12%)	1 (0.88%)
Took Other Vitamins			
Yes	8047 (99.06%)	7958 (98.89%)	89 (1.11%)
No	76 (0.94%)	75 (98.68%)	1 (1.32%)
Took Worm Treatments			
Yes	8105 (99.78%)	8015 (98.89%)	90 (1.11%)
No	18 (0.22%)	18 (100.00%)	0 (0.00%)
Mother’s Working Status			
Yes	1833 (22.57%)	1810 (98.75%)	23 (1.25%)
No	6290 (77.43%)	6223(98.93%)	67 (1.07%)
Mother’s Experience with Domestic Violence			
High	6 (0.07%)	6 (100.00%)	0(0.00%)
Medium	55 (0.68%)	55 (100.00%)	0(0.00%)
Low	8062 (99.25%)	7972 (98.88%)	90(1.12%)
**Household Variables**			
Household Size			
Two	52 (0.64%)	51 (98.08%)	1 (1.92%)
Three	991 (12.2%)	969 (97.78%)	22 (2.22%)
Four	2102 (25.88%)	2075 (98.72%)	27 (1.28%)
Five or Above	4978 (61.28%)	4938 (99.20%)	40 (0.80%)
Source of Drinking Water			
Pipe Born Water	2809 (34.58%)	2775 (98.79%)	34 (1.21%)
Well	3181 (39.16%)	3150 (99.03%)	31 (0.97%)
Other Sources	2133 (26.26%)	2108 (98.83%)	25 (1.17%)
Type of Sanitation Facility			
Flush/ Pour Flush Toilet	7724 (95.09%)	7638 (98.89%)	86 (1.11%)
Pit Latrine	251 (3.09%)	248 (98.80%)	3 (1.20%)
Other	148 (1.82%)	147 (99.32%)	1 (0.68%)
Wealth Index Quintile			
Lowest	2055 (25.3%)	2025 (98.45%)	30 (1.46%)
Second	1662 (20.46%)	1649 (99.22%)	13 (0.78%)
Middle	1509 (18.58%)	1498 (99.27%)	11 (0.73%)
Fourth	1572 (19.35%)	1552 (98.73%)	20 (1.27%)
Highest	1325 (16.31%)	1309 (98.79%)	16 (1.21%)
**Community Variables**			
Type of Residence			
Urban	1304 (16.05%)	1293 (99.16%)	11 (0.84%)
Rural	6294 (77.48%)	6224 (98.89%)	70 (1.11%)
Estate	525 (6.46%)	516 (98.29%)	9 (1.71%)
Province			
Western Province	1525 (18.77%)	1510 (99.02%)	15 (0.98%)
Central Province	1040 (12.8%)	1028 (98.85%)	12 (1.15%)
Southern Province	914 (11.25%)	906 (99.12%)	8 (0.88%)
Northern Province	999 (12.3%)	987 (98.80%)	12 (1.20%)
Eastern Province	922 (11.35%)	904 (98.05%)	18 (1.95%)
North Western Province	883 (10.87%)	875 (99.09%)	8 (0.91%)
North Central Province	552 (6.8%)	550 (99.64%)	2 (0.36%)
Uva Province	574 (7.07%)	567 (98.78%)	7 (1.22%)
Sabaragamuwa Province	714 (8.79%)	706 (98.88%)	8 (1.12%)

Table 3 presents the summary statistics of the continuous variables used in this study, separated by the living status of under five children. The results indicate that the mothers of deceased children had a mean age at first marriage of 23 years, a mean age at childbirth of 29 years, and a mean body mass index (BMI) of 25.39.

**Table 3:** Summary statistics of continuous variables with living status.

Living Status	Variable	Mean	Standard Deviation	Minimum	Maximum
Alive	Mothers Age at First Marriage	23	5	10	43
	Mother’s Age	29	6	14	47
	Mother’s BMI	24.12	4.98	8.01	79.00
Dead	Mothers Age at First Marriage	23	5	14	35
	Mother’s Age	29	5	15	42
	Mother’s BMI	25.39	5.07	14.14	48.79

The mean VIF of 1.43 indicates the absence of high multicollinearity among the independent variables in the study.

### Results of model validation

Tables 4 and 5 show the 2 x 2 classification tables created for the observed and predicted values by the BLRM and BLRIMM, respectively. Table 6 displays the correctly predicted percentage of the living status of children by both models.

**Table 4:** Observed vs predicted observation count by the BLRM.

Observed Living Status	Predicted Living Status		Total
	Alive	Dead	
**Alive**	7942	91	8033
**Dead**	46	44	90
**Total**	7988	135	8123

**Table 5:** Observed vs predicted observation count by the BLRIMM.

Observed Living Status	Predicted Living Status		Total
	Alive	Dead	
**Alive**	7972	61	8033
**Dead**	47	43	90
**Total**	8019	104	8123

**Table 6:** Percentage of correct predictions.

Model	Correctly Predicted Percentage
BLRM	98.31%
BLRIMM	98.67%

Hence, it is clear from the above results that the BLRIMM has correctly predicted 29 more observations than the BLRM, confirming that the use of BLRIMM has provided more accurate predictions than the BLRM.

#### Comparison of correctly predicted percentages

Table 7 presents the results of the comparison of the correctly predicted percentages by the two models, calculated as explained in the methodology section. According to the results, it is clear that the difference in the correct prediction proportion by the two models is significant at a 5% level. Therefore, it can be concluded that the BLRIMM is a better prediction model of U5M than the BLRM.

**Table 7:** Results of comparison of correctly predicted proportions of cases by the two models.

P_1_ = Probability of a correct outcome in the population if predicted by BLRM
P_2_ = Probability of a correct outcome in the population if predicted by BLRIMM
p_1_ = Probability of a correct outcome in the sample if predicted by BLRM
p_2_ = Probability of a correct outcome in the sample if predicted by BLRIMM
H_0_: P_2_ = P_1_
H_1_: P_2_ > P_1_
p_1_ = 0.9831
p_2_ = 0.9867
F_1_. = 8123
F_2_. = 8123
p = 0.9849
variance of p_2_ –p_1_ = 0.0000036617
Z = 1.88 (This is greater than the 5% upper tail value of the standard normal distribution which is 1.64)
p—value = 1—P(x ≤ 1.88) = 0.03 (This is less than 0.05 (5%))
Therefore the null hypothesis, H_0,_ is rejected and it is concluded that BLRIMM gives a higher total correct predictions than the BLRM.

### Results of fixed effects

Table 8 presents the odds ratios and the 95% confidence intervals of the fitted BLRIMM model, to test the association between U5M and the selected variables from the BLRM model.

**Table 8:** Odds ratios and 95% confidence intervals of the BLRIMM parameters.

Variable	Category	Odds Ratio	95% Confidence Interval		
Child was breastfed (ref = Yes)	No	116.74	62.97	216.41	***
Type of Birth (ref = Single)	Multiple	3.73	1.21	11.51	*
Type of Delivery (ref = Normal Delivery)	Other Delivery Types	1.86	1.11	3.12	*
Place of Delivery (ref = While Going to the Hospital/ Other)	Government Hospitals & Specialized Service	0.85	0.02	30.49	
	Other Government Hospitals	0.52	0.01	19.91	
	Private Hospital/ Estate Maternity Home	0.49	0.01	21.63	
	Home	18.75	0.29	>999.99	
Previous Death/ Miscarriage of a Child (ref = No)	Yes	2.27	1.26	4.11	**
Religion (ref = Other Religions)	Buddhist	0.06	0.01	0.50	**
	Hindu	0.05	0.01	0.46	**
	Islam	0.11	0.01	1.20	
	Roman Catholic	0.03	0.00	0.31	**
	Other Christian	1.37	0.06	29.77	
Times Pregnancy TT Received (ref = None)	Once	5.83	0.67	50.97	
	Twice or More	1.99	0.22	18.16	
Household Size (ref = Two)	Three	1.44	0.10	20.36	
	Four	0.56	0.04	7.80	
	Five or More	0.36	0.03	4.99	
Mother’s BMI		1.05	1.00	1.09	*

p-value < 0.05 (*), p-value < 0.01 (**), p-value < 0.001 (***)

According to the results of Table 8, children who were not breastfed were associated with higher odds of U5M (OR = 116.74, 95% CI = 62.97–216.41) compared to children who were breastfed. As this 95% CI is very wide it indicates that the estimated OR is not very accurate. This could be because there are very few dead children and also very few children that were not breastfed. The OR and 95% CI will be accurate mainly for large samples. Children whose type of birth is multiple birth were 3.73 (95% CI = 1.21–11.51) times more likely to die before reaching their fifth birthday than otherwise. Children who were born by other delivery methods opposed to normal child delivery method were associated with higher odds of U5M (OR = 1.86, 95% CI = 1.11–3.12). Children, whose mother had a previous death or miscarriage of a child, have 2.27 (95% CI = 1.26–4.11) times higher odds of dying before reaching their fifth birthday compared to otherwise. The odds ratio of U5M was observed to be low among children, whose mother is a Buddhist (OR = 0.06, 95% CI = 0.01–0.50), Hindu (OR = 0.05, 95% CI = 0.01–0.46) or Roman Catholic (OR = 0.03, 95% CI = 0.00–0.31) compared to “Other Religions” category. The BMI is a continuous variable. This variable has been fitted to the model assuming that. Increase in mother’s BMI by one unit was associated with increasing odds of U5M (OR = 1.05, 95% CI = 1.00–1.09). As the lower limit of its 95% CI is 1.00 the OR is only marginally significant.

### Results of the random effect

Table 9 displays the estimates of the covariance parameter. This indicates the variance among clusters or the extent to which the clusters differ from one another, confirming the presence of a cluster effect in the study data [31].

**Table 9:** Covariance parameter estimates.

Covariance Parameter	Subject	Estimate	Standard Error	P- Value
Intercept	Cluster	0.8231	0.4718	0.0405

The P-value clearly indicates that the covariance parameter or the cluster effect is significant at the 5% level. This is the reason a random effects model is preferred over a simple BLRM.

## Discussion

### Factors associated with U5M in Sri Lanka

The results of the fitted BLRIMM revealed that factors associated with U5M in Sri Lanka include whether or not the child was breastfed, the type of birth, the type of delivery, previous death/miscarriage of a child, religion, and the mother’s BMI.

Breastfeeding for at least six months of life is considered important in protecting the infant’s health [32], and the results of the fitted model further establish this statement. The results show that children who were not breastfed were at a significantly higher risk of U5M compared to those who were. These results are in agreement with studies done in India, Bangladesh, and Ethiopia [18, 33, 34]. However, the very high odds ratio should be interpreted with caution because the confidence interval is very wide.

Multiple births have been a significant factor associated with U5M in Ghana, Ethiopia, and Bangladesh [20, 35, 36]. In the Sri Lankan context, the type of birth was also a significant factor, with children born as part of multiple births having a higher risk of dying before five years compared to singleton births.

The odds of children dying before their fifth birthday are higher among those delivered using other methods such as caesarean, forceps, and vacuum compared to normal delivery. Perera, Assefa, and Amilani [37] showed in a study in Sri Lanka that normal vaginal delivery had a negative association with U5M in univariate analysis. This could be due to the element of risk involved in other methods compared to normal delivery. The results obtained are also in agreement with research done in the USA, Tanzania, and Nigeria [38–40].

The results of the fitted model revealed that the odds of U5M of children whose mothers had a previous death or miscarriage are significantly higher than those who did not. A previous study explains one possible reason for this as the long-term psychological effect of child death on parents, which can result in poor nutrition and inadequate essential healthcare for surviving children [41]. Another reason could be a similar genetic health condition or disease that caused the death of a previous child affecting the health of surviving children and leading to their death. Furthermore, previous miscarriages may indicate a pregnancy complication, and even though the complication did not result in the miscarriage of the child, it may still affect the health of living children and lead to their death. The results of research conducted in Nepal and Bangladesh [19, 41] support the findings of this study regarding this factor.

Although religion was a significant predictor in the fitted model, some categories within this variable, such as children of mothers who were Muslim or other Christians, were not significant. However, categories such as children whose mothers were Buddhist, Hindu, or Roman Catholic were significant predictors of the "Religion" variable, and the odds of U5M in these significant categories were lower than those of children whose mothers belong to the "Other Religions" category. These "Other Religions" could include minority religions like Bhai’s faith, Sikhism, which have come from India, and those without a religion, such as Atheists. There are various practices followed by families belonging to different religions regarding a new-born child, including feeding and nutritional practices and overall lifestyle. These practices can include differences in when and how long breastfeeding takes place, when other liquids and solids are introduced to the diet, and what is fed to the child as they grow. For example, Hindu and Buddhist children are typically not given most meats, Muslim children are not given pork, and Christian children have no food restrictions. With regards to lifestyle, all Christians, including Roman Catholics, baptize their newborn children, and male Muslim children are circumcised. Young children of these religions are exposed to visiting churches/mosques at an early age. Westernized nursery schools and Convent-based schools usually teach Christianity to young children, while Temple (Buddhist/Hindu) or Mosque-based schools teach their respective religions. Literature suggests that the relationship between religion and child survival can be attributed to religion-related differences in the use of health care services [42]. Additionally, this can be due to the diversity and significant differences in types of food and nutrition intake and lifestyle practices among different religions, which can directly affect the variation of U5M among religions.

The BMI cut-offs for Asians are 23–24.9 kg/m2 as overweight and ≥25 kg/m2 as obese, as it has been stated that Asians have different associations between BMI, percentage of body fat, and health risks compared to Europeans [43]. The results of the fitted BLRIMM model show that the likelihood of U5M increases with increasing BMI of the mother. A document based on the management of diabetes during pregnancy states that "being South Asian and pregnant, places women in Sri Lanka at a higher risk for diabetes during pregnancy, so universal screening using a diagnostic test is recommended for all Sri Lankan women" [44]. Some studies have shown a relationship between high maternal pre-gestational BMI and high gestational weight gain, leading to increased risk of insulin resistance, obesity, and type 2 diabetes in the offspring [45]. Previous research also suggests that maternal obesity has adverse effects on infants, with the odds of infant mortality being twice as high among obese women compared to lean women [46]. A study conducted in 2017 across 9 Asian countries shows that maternal overweight is associated with neonatal mortality [47]. Further studies on child mortality and its association with maternal obesity suggest a positive correlation between the mother’s increasing BMI and infant and neonatal mortality, due to increased risk of labor complications, macrosomic disorders in infants, and increased susceptibility to diseases and impaired health during the life course among children with prenatal exposure to maternal obesity [48–53]. Hence, efforts should be made to raise awareness among mothers to maintain a healthy lifestyle, with a suitable BMI, before, during, and after pregnancy.

Even though numerous research studies have been conducted on U5M in Sri Lanka and globally, suggesting that factors such as wealth index quintile, type of place of residence, mother’s education, sex of the child, sources of drinking water, type of sanitation facility, birth order, marital status, and mother’s age at childbirth contribute to U5M, these did not emerge as factors in the Sri Lankan context based on the study sample considered.

Since the survey was conducted in 2016, the current rate of U5M has decreased compared to what was recorded at that time, as evidenced by the 2020 record of 6.9 deaths per 1000 live births in Sri Lanka. It is evident that advancements have been made over the 4-year period. These changes include increased access to clinics for pregnant mothers, more programs to educate parents, higher levels of education among mothers, the availability of numerous reports and papers on U5M and its factors, and the widespread availability of the internet in major languages such as English, Sinhala, and Tamil, among others.

### Use of multilevel modeling

**Fig 2:**
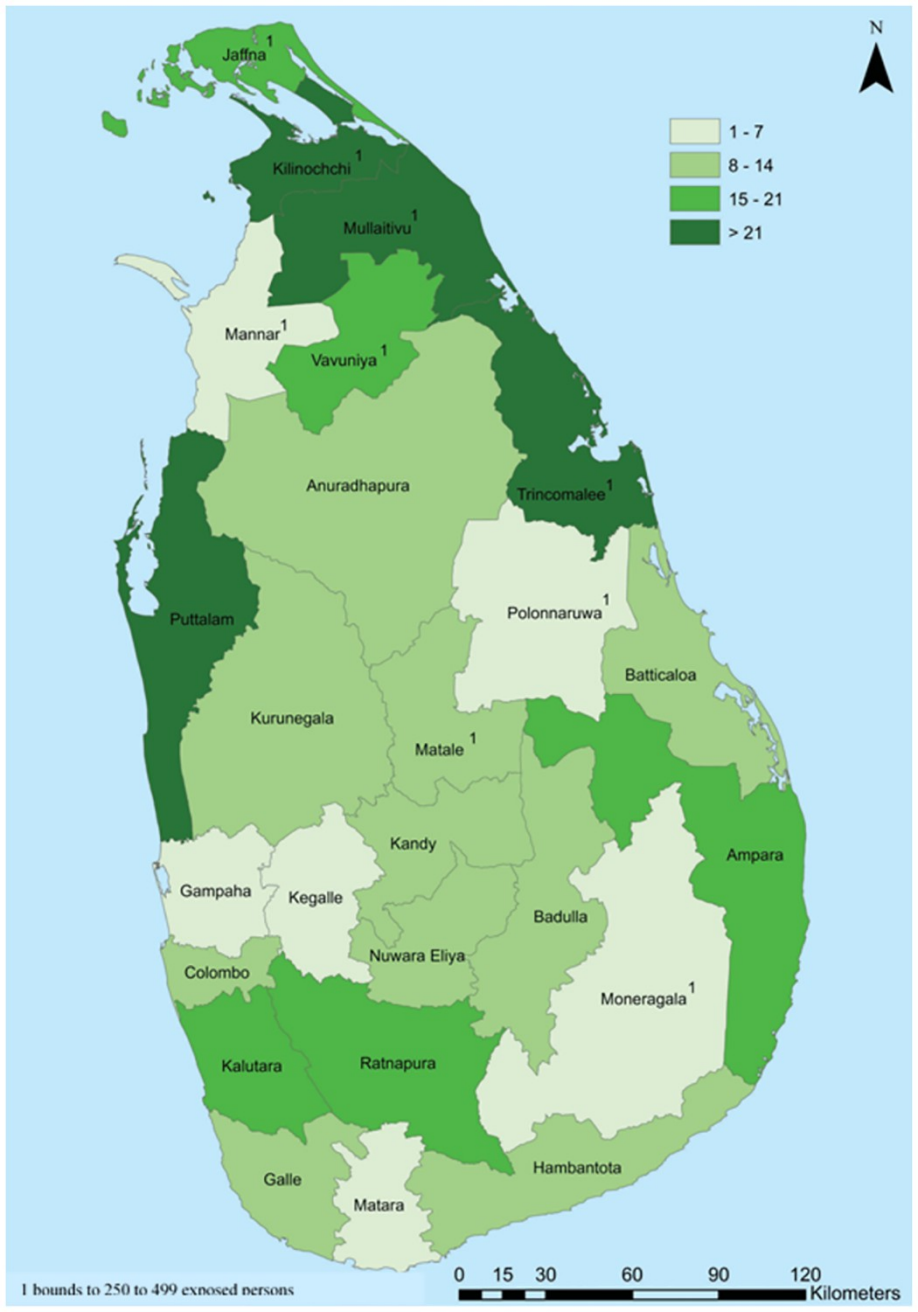
Spatial distribution of U5M rates by districts of Sri Lanka using data from the 2016 Sri Lanka demographic and health survey. Republished from [54] under a CC BY license, with permission from DCS, original copyright [2017].

The covariance parameter estimate of the BLRIMM shows that the variance among the clusters is not zero and there is a significant variance among the clusters. This highlights the importance of fitting a BLRIMM that accounts for the clustering effect, rather than just fitting a model that does not take this feature of the sample design into account. This is supported by the percentage of correctly predicted living status of children by the two models, where the BLRIMM has a higher percentage of correct predictions compared to the BLRM. Fig 2 shows the spatial distribution of U5M rates by district in Sri Lanka. It is evident that the U5M rates vary from district to district, confirming the significant cluster variance resulting from the multilevel analysis. These differences could have arisen due to factors such as wealth within the district, health facilities within the district, the number of pediatricians in the district, etc.

### Recommendations

In 2017, the government of Sri Lanka developed "The Maternal and Newborn Health Strategic Plan (MNH SP) (2017–2025) of Sri Lanka" with a vision of ensuring "a country in which there are no preventable deaths of mothers, fetuses, and newborns, where every pregnancy is planned and wanted, every birth is celebrated, and women, babies, and children survive, thrive, and reach their full potential" [55]. The findings of the research study suggest that further interventions are needed to raise awareness among pregnant mothers about the importance of breastfeeding. This can align with the major activity on "Protect, Promote, and Support Breastfeeding Practices in All Settings" in the MNH SP. Understanding when and where to use alternative delivery methods, and improving the facilities to reduce U5M when other delivery methods are used, is important. The MNH SP has set "Implement a Uniform Sustainable, Quality Assurance System on Pre-Pregnancy Care, ANC, Intra Natal Care (INC), Post Natal Care (PNC), and Newborn Care at Institutional and Field Level" as a major activity, and this program can further examine methods of improving the quality of alternative delivery methods to reduce U5M. Furthermore, pregnant mothers, their partners, and other responsible family members must be educated to take extra measures to ensure the safety of the mother and child during pregnancy, especially with multiple children and to prevent miscarriages. Encouraging mothers who have experienced a previous death or miscarriage of a child to use frequent visits to the ANC services and other health services related to pregnancy and childbirth can be beneficial for the child’s survival. As the results indicate that the odds of U5M for mothers belonging to Buddhist, Hindu, or Roman Catholic religions is lower compared to other religions, targeting the "other religion" category with appropriate and timely interventions may yield larger gains. These recommendations can be added as part of the major activity on "Develop a System to Support Informed Decision Making for the Management to be Carried Out on the Baby or Mother During Pregnancy, Delivery, and Post-Partum Period" in the MNH SP. In addition, raising awareness among women about the importance of a healthy dietary pattern to maintain a healthy BMI, proper family planning, and practicing a healthy lifestyle before, during, and after pregnancy is essential. These issues can be addressed by improving existing healthcare programs at maternity clinics, women’s wellness centers, and family planning units, and by establishing these centers and units in all regions in the country, which can increase access to these services and programs for many women and pregnant mothers.

### Limitations of the study

This study was a cross-sectional study that used only 2016 DHS data, and therefore the results only depict the situation of U5M during the 2011–2016 time period. The trend of U5M can be further examined by incorporating multiple DHS datasets. However, the data collected in this study were based on self-reported responses from the respondents, which may result in inaccurate or misleading information. Additionally, as the data collected were based on children born within five years prior to the time of the survey, recall bias may have affected the accuracy of the responses in the study variables. Another limitation of the study is that, although there are many explanatory variables involved in the complex models, the observations in the dataset are limited, which could result in overfitting of the models and incorrect predictions. Some important and significant variables found in other studies were not found to be significant in this study, which may be due to the insufficient sample size and lack of power to detect these variables as significant. Finally, some significant variables from previous studies were not included in the data set because they were not available.

## Conclusions

The aim of this study was to identify the prognostic factors associated with U5M in Sri Lanka. The results showed that not breastfeeding, having multiple births, using other delivery methods instead of normal delivery, having a previous death or miscarriage of a child, and having a higher mother’s BMI increases the odds of U5M in Sri Lanka, while being a Buddhist, Hindu, or Roman Catholic reduces the odds. To reduce U5M, it is important to strengthen the ANC services and encourage frequent visits by pregnant mothers, especially those who have experienced a previous miscarriage or child death. Interventions targeting "other religions" could also be effective. The study also found a significant clustering effect of U5M in different geographical regions, and the BLRIMM was found to predict the results more accurately than the BLRM. These findings can be used by relevant authorities to make informed decisions for policy formulation, plan effective health programs, raise awareness about the prognostic factors, encourage the use of ANC facilities and other health services, and promote ways to reduce U5M in Sri Lanka, which could potentially save lives in the future.
